# Conducting a multi‐country online alcohol survey in the time of the COVID‐19 pandemic: Opportunities and challenges

**DOI:** 10.1002/mpr.1875

**Published:** 2021-05-05

**Authors:** Carolin Kilian, Jürgen Rehm, Peter Allebeck, Miroslav Barták, Fleur Braddick, Antoni Gual, Silvia Matrai, Benjamin Petruželka, Vladimir Rogalewicz, Ingeborg Rossow, Bernd Schulte, Mindaugas Štelemėkas, Jakob Manthey

**Affiliations:** ^1^ Institute of Clinical Psychology and Psychotherapy Technische Universität Dresden Dresden Germany; ^2^ Institute for Mental Health Policy Research Centre for Addiction and Mental Health Toronto Ontario Canada; ^3^ Dalla Lana School of Public Health University of Toronto Toronto Ontario Canada; ^4^ Faculty of Medicine Institute of Medical Science University of Toronto Toronto Ontario Canada; ^5^ Campbell Family Mental Health Research Institute Centre for Addiction and Mental Health Toronto Ontario Canada; ^6^ Department of Psychiatry University of Toronto Toronto Ontario Canada; ^7^ I.M. Sechenov First Moscow State Medical University (Sechenov University) Moscow Russian Federation; ^8^ Department of Global Public Health Karolinska Institute Stockholm Sweden; ^9^ Department of Addictology First Faculty of Medicine and General University Hospital in Prague Charles University Praha Czech Republic; ^10^ Clínic Foundation for Biomedical Research (FCRB) Barcelona Spain; ^11^ Clinical Addictions Research Group (GRAC‐GRE) Psychiatry Department Neurosciences Institute Hospital Clínic University of Barcelona Barcelona Spain; ^12^ Institut d’Investigacions Biomèdiques August Pi i Sunyer (IDIBAPS) Barcelona Spain; ^13^ Norwegian Institute of Public Health Oslo Norway; ^14^ Centre for Interdisciplinary Addiction Research University Medical Center Hamburg‐Eppendorf Hamburg Germany; ^15^ Health Research Institute Faculty of Public Health Lithuanian University of Health Sciences Kaunas Lithuania; ^16^ Department of Preventive Medicine Faculty of Public Health Lithuanian University of Health Sciences Kaunas Lithuania; ^17^ Department of Psychiatry Medical Faculty University of Leipzig Leipzig Germany

**Keywords:** epidemiology, methodology, representativeness, substance use, surveys

## Abstract

**Objectives:**

This contribution provides insights into the methodology of a pan‐European population‐based online survey, performed without external funding during the COVID‐19 pandemic. We present the impact of different dissemination strategies to collect data from a non‐probabilistic convenience sample and outline post‐stratification weighting schemes, to provide guidance for future multi‐country survey studies.

**Methods:**

Description and comparison of dissemination strategies for five exemplary countries (Czechia, Germany, Lithuania, Norway, Spain) participating in the Alcohol Use and COVID‐19 Survey. Comparison of the sample distribution with the country's actual population distribution according to sociodemographics, and development of weighting schemes.

**Results:**

The dissemination of online surveys through national newspapers, paid social media adverts and dissemination with the support of national health ministries turned out to be the most effective strategies. Monitoring the responses and adapting dissemination strategies to reach under‐represented groups, and the application of sample weights were helpful to achieve an analytic sample matching the respective general population profiles.

**Conclusion:**

Reaching a large pan‐European convenience sample, including most European countries, in a short time was feasible, with the support of a broad scientific network.

## 1 INTRODUCTION

The severe acute respiratory syndrome coronavirus 2 (SARS‐CoV‐2) and the resulting COVID‐19 pandemic, which overwhelmed the global population in 2020, has posed unique challenges to the research community. With the rapidly increasing numbers of infections and deaths worldwide and political measures locking down entire countries across the globe in early 2020, scientific studies were urgently needed not only on the front line of disease control and treatment, but also in monitoring public health issues (Clay & Parker, 2020; Holmes et al., 2020; Rehm, Kilian, Ferreira‐Borges, et al., 2020). The exceptional situation created as a result of the pandemic heightened the need for international collaboration and challenged the ability to take rapid, timely action without lengthy research planning, while maintaining the highest standards of research.

In order to trace the immediate impact of the COVID‐19 pandemic on alcohol consumption from the very beginning, a collaboration of European alcohol researchers joined forces to carry out a survey in 22 European countries, on short notice and without external funding. We set up a short online survey, which provided a timely and low‐cost option to gather relevant information on alcohol and other substance use. The survey was completely anonymous and thus facilitated ethical assessment on the one hand, and offered low‐threshold participation for interested people on the other hand. Despite these advantages of online surveys, there are major limitations to be considered, most notably the question of statistical representativeness for the target population (Greenacre, 2016; Kruskal & Mosteller, 1979; Wright, 2006; for a recent discussion on the representativeness of alcohol surveys, see; Mäkelä, 2021; Rehm, Kilian, & Manthey, 2021; Rehm, Kilian, Rovira, et al., 2021). In this report, we present the strategies employed in the design and execution of the project. We discuss opportunities and challenges with the methodology used, and particularly aim to assess the impact of different dissemination strategies in order to obtain population‐based convenience samples from various countries. We first present the objectives of the Alcohol Use and COVID‐19 survey, followed by an overview of dissemination strategies of exemplary countries and an evaluation on the population covered. Finally, we discuss our approach and suggest directions for future multi‐country studies.

## 2 METHODS

### 2.1 Objectives and implementation of the alcohol use and COVID‐19 survey

Our research focused on changes in alcohol consumption, since alcohol use poses a major risk factor for the burden of disease in Europe (GBD 2017 Risk Factors Collaborators, 2018; World Health Organization, 2018). Alcohol use is closely linked to poor physical and mental health outcomes (Rehm et al., 2017), and is likely to change in stressful times, such as the COVID‐19 pandemic (Clay & Parker, 2020; Rehm, Kilian, Ferreira‐Borges, et al., 2020). With the objective to collect self‐reported changes in alcohol use in order to answer pre‐registered hypotheses (see the study protocol Kilian et al., 2020), we designed a rapid pan‐European online survey via the open source tool LimeSurvey (LimeSurveyGmbH, 2020). The questionnaire was developed in English (Kilian, 2020c) and subsequently translated into other languages with support from the existing network of the DEEP SEAS Contract (Developing and Extending Evidence and Practice from the Standard European Alcohol Survey–www.deep‐seas.eu), as well as from other health care professionals and alcohol researchers supporting the research activity (for details, see Data S1). Once the first surveys became publicly available at the end of April, further researchers contacted our study group and asked to join the collaboration in order to carry out the survey in their country. By mid‐May, 3 weeks after launching the project, 21 translations of the survey were available via our study homepage (www.covid19‐and‐alcohol.eu). The survey was distributed in a decentralized manner, using non‐probabilistic convenience sampling, with each national partner taking responsibility for dissemination in their country (for details, see Data S2 or Kilian, 2020a) in two overlapping but independent survey waves. The first survey wave, which we are primarily referring to in this report, covered 22 countries located in Western, Southern, Northern, and Central Europe (24th April to 22nd July, 2020), while the second covered additional 17 countries of Eastern Europe and Central Asia (9th July 2020 to 15th January 2021).

**FIGURE 1:**
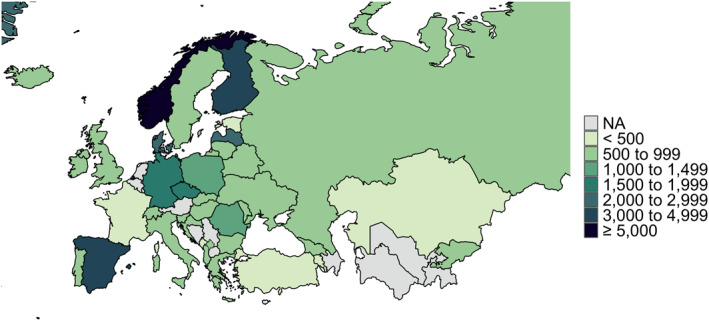
Total number of respondents by country. Grey/NA: not covered in the final sample. Russia and Ukraine were part of both survey waves. Disclaimer: The map represents the territorial borders under the consideration of the United Nations Security Council resolution 1244 from 10th June 1999 (Kosovo) and the United Nations General Assembly

At the end of the first data collection period (22nd July 2020), there had been 125,936 visits to the study link and we achieved the minimum target sample size of *n* = 402 per country (for sample size calculations, see Kilian et al., 2020) everywhere except in the Netherlands (*n* = 109) and France (*n* = 391). Since the number of responses was far below the target sample size in the case of the Netherlands, these responses were removed from the final database. The completion rate, that is, the proportion of all respondents who started responding to the survey questions and who went on to complete the survey, was 75.2%. In total, we had 40,064 complete and valid responses in the first survey wave, ranging between *n* = 391 replies in France to *n* = 17,092 in Norway, and additional 11,856 responses collected during the second wave (range: *n* = 347 in Estonia to *n* = 1998 in Latvia). The number of respondents by country are mapped in Figure 1. For this methodological report, we have selected five countries, all of which reached a remarkable sample size while employing quite different dissemination strategies: Czechia, Germany, Norway, and Spain (first data collection), as well as Lithuania, which took part in the second survey wave, and for which we faced challenges in data collection.

### 2.2 Dissemination strategies used by country

Next, we present the dissemination strategies used in each country, which we will then link to the sample size in order to identify effective strategies. We elaborate on the most effective strategies, which we understand to be dissemination strategies, which resulted in a steep increase in the number of participants (i.e., more than 150 participants within a single week). Furthermore, the direct costs of the strategies employed and their impact duration, that is, the period following the implementation of a strategy in which responses were registered, were used for evaluation. We would like to note that the ‘we’ used in the following paragraphs (i.e., in the presentation of country‐specific strategies) does not denote the entire group of authors, but rather those responsible for the respective country.

### 2.3 Norway

On 14th May 2020 the Norwegian translation of the survey was available online. The next day, we started contacting larger national media, promoted the pan‐European survey, and offered an interview with the Norwegian researcher working on the study, including more detailed information about the survey. On May 18th, on our third attempt, a large national newspaper (Dagbladet) showed interest, and on May 20th, the newspaper published an interview to encourage people to take part in the survey (Braseth, 2020). A direct link to the survey was provided in the online version. On the same day, we also encouraged the visitors of the Norwegian Institute for Public Health's website and social media to participate in the survey (Folkehelseinstituttet, 2020).

### 2.4 Spain

*S*urvey dissemination in Spain started on 24th April 2020. Initially, the link to the survey was shared on different social media channels and published on the institutions' website. Two weeks later, the survey received additional support from the Spanish National Plan on Drugs, which also distributed the survey through their social media accounts and website. The Spanish National Plan on Drugs is a governmental research and public health body, under the direction of the Ministry of Health, operating at national level, and addressing citizens, professionals, and politicians.

### 2.5 Germany

The dissemination of the German survey started on 24th April 2020 mainly by the distribution through professional networks and student mailing lists from various universities as well as private social media accounts. Additionally, a paid Facebook ad was placed on June 10th to reach underrepresented populations. This was possible by leveraging the services of an online provider to place social media adverts on Facebook, allowing us to address specific target groups by gender and age.

### 2.6 Czechia

In Czechia, where dissemination also started on 24th April 2020, we used the website and Facebook page of the Public Health Centre for Alcohol‐related Harm (Charles University in Prague). Additionally, social contacts were employed to reach different respondent groups (small and middle‐size communities, young and elderly, different regions). The survey was further distributed using mail directories of the Charles University and regional university students. Moreover, we addressed academic colleagues in different universities across Czechia. Additionally, efforts were supported by professional organisations in the field of addiction, including the National Drug Coordinator, the Office of the Government of the Czech Republic, and by the Ministry of Health. A press release was published at the Charles University followed by the Czech Press Agency (ČTK) and printed in several local newspapers. In the beginning of May, we placed public‐health related texts free of charge into advertisement screens of regional public transport vehicles. Information about the survey and the survey link was included in a short video. To increase the response rate in Prague, in mid‐May we asked respective authorities of the City of Prague to publish a press release about the survey on their website (‘Alkohol a COVID 19,’ 2020). Additionally, an interview was published in the online magazine of Charles University (iForum, Uhlíková, 2020) at the end of May.

### 2.7 Lithuania

Lithuania participated in the second survey wave, with dissemination starting on 4th August 2020, when the first message on the Facebook page of the Health Research Institute (HRI, Kaunas) was posted, asking people interested in the survey to visit the study homepage. The message was reposted on September 3rd. Furthermore, the information about the survey was circulated with the help of the Lithuanian Ministry of Health (a post on the Ministry's Facebook page), Lithuanian University of Health Sciences (Facebook post and an advert on the university's website), among local professional and student email networks, as well as in private accounts on Facebook and a paid‐for Facebook ad. It is important to mention that these first strategies used a link that directed people from the study homepage to the survey. This approach was taken because the majority of the countries participating in the second wave of the survey had more than one commonly spoken language (e.g., Lithuanian, Russian) and at the same time had diverse currencies and income distributions. However, at the end of October, this strategy was changed and the website link was replaced by a direct link to the survey (as in the first survey wave). A new post on the Facebook page of HRI was released and immediately boosted to a paid‐for ad using a direct web link to the Lithuanian version of survey. Furthermore, the advert on the university's website was also updated using a direct link to the Lithuanian survey.

### 2.8 Post‐stratification weighting procedure

First of all, the Alcohol Use and COVID‐19 Survey was not planned as a probabilistic survey, that is, trying to establish representativeness via sampling strategies and minimal non‐response (see Kruskal & Mosteller, 1979; Rehm, Kilian, Rovira, et al., 2021; see also study protocol; Kilian et al., 2020). Moreover, the main aim was not to establish population prevalence, but to test pre‐registered hypotheses. Thus, the project was designed to cover as many adults as possible to constitute a large non‐probabilistic convenience sample. However, in order to evaluate how sub‐populations vary according to gender, age, and educational attainment, we compared key sociodemographic aspects of our sample to the actual population distribution from EUROSTAT (EUROSTAT, 2020) for the five exemplary countries.

We further computed survey weights depicting the inverse probability for taking the survey, which were calculated for 18 strata per country, based on the gender, age group, and educational attainment (i.e., post‐stratification adjustment). In order to avoid overweighting single observations, a maximum weight of 10 was set (i.e., with a weight of maximum of 10, an observation can be counted up to 10 times compared to an observation with a weight of 1) and strata were collapsed within each gender if this limit was exceeded (for details, see Kilian, 2020b). Collapsing the strata was done stepwise: first, the stratum of an excessive weight (>10) was collapsed with its direct stratum neighbour (e.g., women with primary education aged 18–34 and 35–54 years), and subsequently, if the resulting weight was still above the limit, with three stratum neighbours (e.g., women with primary and secondary education aged 18–34 and 35–54 years) or even with six stratum neighbours (e.g., women with primary and secondary education of all ages).

## 3 RESULTS

**FIGURE 2:**
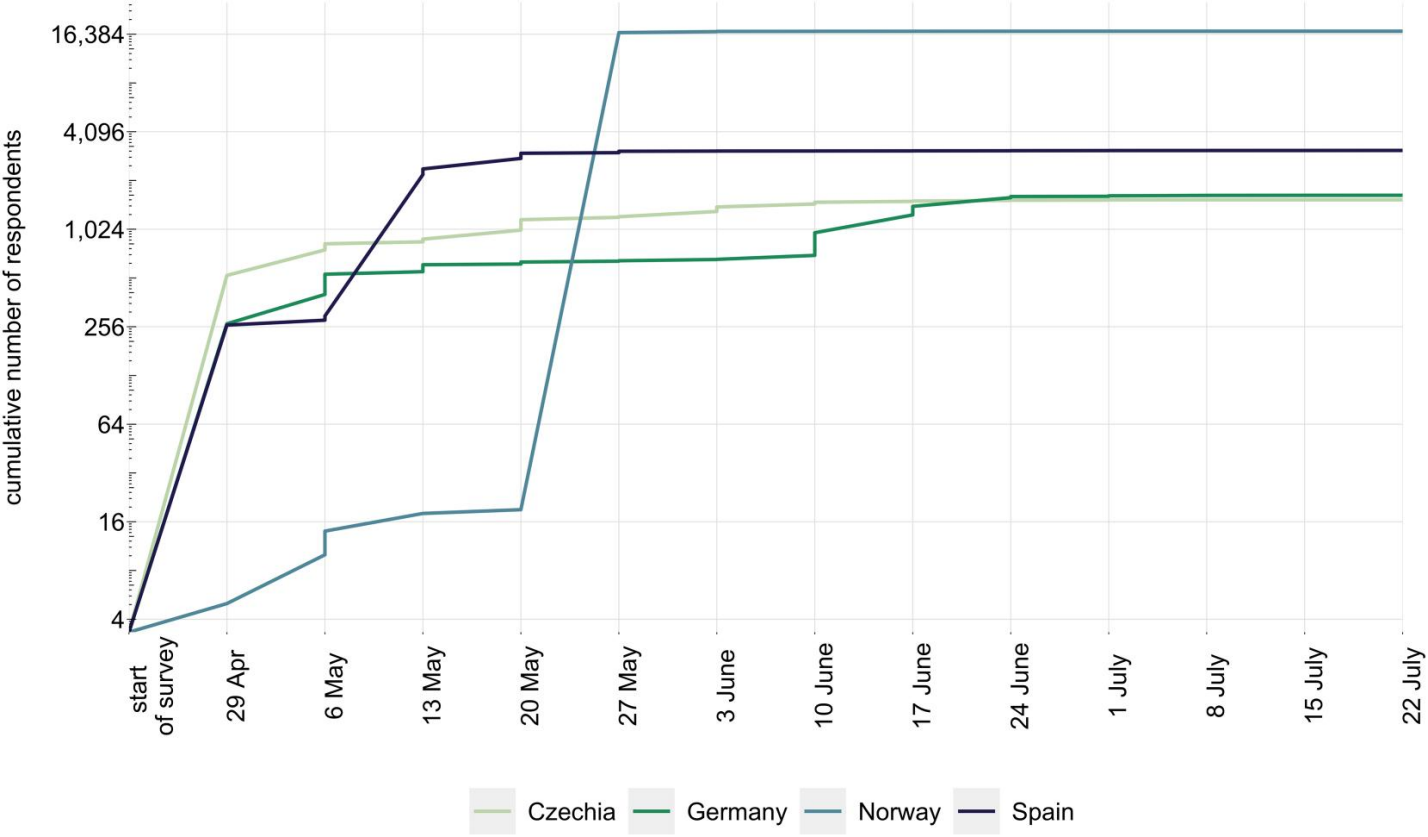
Cumulative number of valid responses for Czechia, Germany, Norway and Spain are displayed by week of data collection (in the year 2020). Dates reflect weekly point estimates. Numbers for Lithuania are not presented since the country participated in the second survey wave (data collection: 4th August, 2020 to 12th December 2020)

Information on the number of respondents by survey week for four of the selected countries which were part of the first survey wave are plotted in Figure 2. Steep increases in the number of respondents were clearly visible in the weeks from May 6th to May 13th for Spain, May 20th to May 24th for Norway, and June 10th to June 17th for Germany. In Czechia multiple steep increases can be observed, although clearly smaller than in other countries. Steep rises in sample sizes can be linked to particular dissemination measures adopted in these countries. Table 1 gives an overview of the respective strategies, their costs, the number of recorded responses, along with the period during which clearly more responses were registered, and the socio‐demographic characteristics of the population reached.

**TABLE 1:** Key dissemination strategies and their output by country

	Type of strategy	Cost of strategy^a^	Number of responses (% of Total country sample)^b^	Impact period^c^	Socio‐demographic characteristics of the sample compared to the general population
Czechia					
#1	Professional networks, students mailing lists, press release (Czech press agency), personal contacts, support by the Ministry of Health	0 EUR	532 (34.2%)	1 week	Oversampling of women, middle‐aged adults, respondents with higher educational attainment
#2	Advertisement in public transport	0 EUR	301 (19.4%)	1 week	Oversampling of women, middle‐aged adults, respondents with higher educational attainment
#3	Post on the official city of Prague website	0 EUR	283 (18.2%)	1 week	Oversampling of women, young adults, more respondents with secondary education as before, but still a high proportion of those with higher education
#4	Interview on the survey for a University magazine	0 EUR	279 (17.9%)	2 weeks	Oversampling of women, young adults, more respondents with secondary education as before, but still a high proportion of those with higher education
Germany					
#1	Professional networks, students mailing lists, institution's website and personal social media posts	0 EUR	706 (42.6%)	6 weeks	Oversampling of women, young adults, respondents with higher educational attainment
#2	Paid Facebook ad	500 EUR	953 (57.4%)	4 weeks^d^	Oversampling of respondents with higher educational attainment
Norway					
#1	National newspaper, institution's website and social media posts	0 EUR	16,996 (99.4%)	Majority within 2 days^e^	Oversampling of women, middle‐aged adults, and respondents with higher educational attainment
Spain					
#1	Institution's website and social media posts	0 EUR	298 (9.5%)	2 weeks	Oversampling of women, middle‐aged adults, and respondents with higher educational attainment
#2	Website and social media posts supported by the Spanish National Plan on Drugs	0 EUR	2717 (86.6%)	2 weeks	Oversampling of women, young and middle‐aged adults, respondents with higher educational attainment
Lithuania					
#1	Professional networks, student mailing lists, personal contacts, support by the Ministry of Health, ad on the University website, a paid Facebook ad for 8 days.	10 EUR	164 (28.4%)	12 weeks	Oversampling of women, respondents with higher educational attainment
#2	Dissemination of direct link to Lithuanian survey via paid Facebook ad, updated University website ad.	45 EUR	413 (71.6%)	6 weeks	Oversampling of women, young adults, respondents with higher educational attainment

^a^ Costs of strategy reflect direct costs only, ‘in‐house’ costs such as personnel costs are not included.

^b^ Estimated number of respondents based on the week of registered participation; possibility that people participated due to previous dissemination strategies cannot be discounted.

^c^ Impact period is defined as the period after a strategy was implemented in which there was an increase in participation either characterised by a spike in the number of participants (number of participants at least doubled) or there was a steady increase of participants per week after the implementation of the strategy. Number of participants presented in the table refers to this impact period.

^d^ The advertisement was shown to various target groups over a period of four weeks.

^e^ Sixteen thousand five forty‐eight responses (97.3%) were registered in just 2 days, with a further 448 responses registered the following week.

In Norway, after the introduction of the two key dissemination strategies the response rate increased substantially in a very short period of time. Until May 22nd, around 35,000 entries were registered, and this huge response resulted in technical problems with server capacity. As a consequence, many respondents found that the time required to answer the survey was unacceptably long or that survey completion was impossible. Of the 35,000 entries registered, over 18,000 entries to the Norwegian survey were considered invalid, and around a third of these reflected incomplete responses (i.e., people who started responding to the survey questions but did not complete it). Given these problems, we ended up with 16,500 complete responses from Norway. In the following weeks, an additional 544 valid responses were registered, whereupon relatively few responses added to the survey until the closing date of June 30th. While information on the exact time or the location of respondents was not collected, it seems most likely that the massive spike in survey responses within a very short period can be attributed to the newspaper interview including the link to the online version of the survey. Notably, no other strategies to attract survey respondents were employed around that time in Norway.

In Spain, social media channels and local institutional websites managed to elicit 300 responses within the first 2 weeks. Disseminating the survey through social media and including it on the websites of the Spanish National Plan on Drugs led to a large interest in the survey, with more than 2700 additional responses collected within the next 2 weeks. As with Norway, no other strategies were considered in Spain.

In Germany, we reached about 700 people within the first 6 weeks, of which a disproportionate number were young and reported higher education. After the paid‐for Facebook ad was placed, between 200 and 250 people completed the survey on a weekly base in the following 4 weeks, resulting in more than 1600 participants in the end of the campaign. The strategy was able to partially balance out the initially skewed distribution of participants in Germany with regard to gender and age.

In Czechia, the mix of dissemination efforts used during the first week resulted in more than 500 responses. The short video advertisement presented in the public transport, which was displayed 1,272,600 times in 17 Czech regional cities, resulted into 301 additional participants within 1 week. The press release that targeted particular citizens in Prague lead to an estimated number of about 280 new respondents within the following week, while the interview published in the online Charles University magazine contributed by approximately 100 responses. Taken together, dissemination efforts in Czechia resulted in more than 1500 responses.

In Lithuania, all strategies employed before October resulted in a total of only 164 responses over a period of almost 3 months. However, after replacing the link that redirected people to the study website with the direct link to the Lithuanian survey, more than 400 completed responses were registered over the next 6 weeks. We think that the obstacles we have encountered in Lithuania may have been due to the additional number of clicks required to access the survey via the study website, a language barrier (the study page being in English), and some later changes on the study homepage (additional information appeared on the website which pushed the link to the Lithuanian survey further down the page). The change in dissemination strategy of replacing the general homepage link to the direct link appeared to be a major turning point, leading to increases in the number of responses.

Altogether, by far the highest number of respondents was achieved in Norway, which was largely due to an interview with the Norwegian researcher in a national newspaper, coupled with a direct link to the survey in the online version of the article. Spain achieved a large sample size with the support of the Ministry of Health. Germany relied on paid social media adverts (see Table 1 for costs), while a wide range of strategies, from adverts in public places to a published interview on the survey, were applied in Czechia. All participating institutions contributed with ‘in‐kind’ resources not included in Table 1, such as personnel costs or infrastructure.

### 3.1 Coverage of the population by the sample data

The unweighted and weighted data that accounted for the known sampling bias according to the three key sociodemographic characteristics (i.e., gender, age, and educational attainment; for detailed information, see Kilian, 2020b) in comparison to the actual population of the four countries is presented in Table 2.

**TABLE 2:** Socio‐demographic characteristics of the survey population and the country population according to EUROSTAT

		EUROSTAT population	Survey population		
			Unweighted	Weighted	*N*
Czechia (*N* = 1555)					
Gender (%)	Women	50.1	69.3 (66.9, 71.5)	50.0 (45.5, 54.6)	1077
	Men	49.9	30.5 (28.3, 32.9)	49.9 (45.3, 54.4)	475
	Other	NA	0.2 (0.1, 0.6)	0.1 (0.0, 0.3)	3
Age groups (%)	18–34 years	26.3	45.7 (43.3, 48.2)	26.3 (23.3, 29.6)	711
	35–54 years	40.8	42.3 (39.9, 44.8)	40.8 (36.6, 45.2)	658
	≥55 years	32.9	12.0 (10.4, 13.7)	32.9 (28.0, 38.2)	186
Educational attainment (%)	Primary	9.4	2.1 (1.5, 3.0)	6.6 (4.7, 9.3)	33
	Secondary	69.8	33.1 (30.8, 35.5)	62.5 (58.4, 66.4)	515
	Higher	20.8	64.8 (62.3, 67.1)	30.9 (27.5, 34.5)	1007
Germany (*N* = 1659)					
Gender (%)	Women	49.8	51.1 (48.7, 53.5)	49.4 (46.2, 52.5)	848
	Men	50.2	48.0 (45.6, 50.4)	49.7 (46.5, 52.8)	796
	Other	NA	0.9 (0.5, 1.5)	1.0 (0.5, 1.9)	15
Age groups (%)	18–34 years	27.5	36.2 (33.9, 38.6)	27.6 (25.1, 30.3)	601
	35–54 years	37.8	42.7 (40.4, 45.1)	37.9 (35, 40.9)	709
	≥55 years	34.7	21.0 (19.1, 23.1)	34.5 (31.3, 37.8)	349
Educational attainment (%)	Primary	16.0	14.8 (13.1, 16.6)	16.0 (14.0, 18.2)	245
	Secondary	57.1	27.8 (25.7, 30.1)	57.2 (54.2, 60.1)	462
	Higher	26.9	57.4 (55.0, 59.7)	26.8 (24.7, 29.0)	952
Lithuania (*N* = 577)					
Gender (%)	Women	52.4	17.3 (14.5, 20.6)	46.8 (37.7, 56.1)	100
	Men	47.6	82.5 (79.2, 85.4)	51.5 (42.3, 60.6)	476
	Other	NA	0.2 (0.0, 1.2)	1.7 (0.2, 11.1)	1
Age groups (%)	18–34 years	29.1	56.3 (52.2, 60.3)	40.5 (32.1, 49.5)	325
	35–54 years	36.8	31.9 (28.2, 35.8)	42 (33.1, 51.5)	184
	≥55 years	34.1	11.8 (9.4, 14.7)	17.5 (11.4, 25.8)	68
Educational attainment (%)	Primary	7.8	0.3 (0.1, 1.4)	3.4 (0.9, 12.5)	2
	Secondary	54.8	19.4 (16.4, 22.8)	59.8 (51.6, 67.5)	112
	Higher	37.4	80.2 (76.8, 83.3)	36.8 (29.9, 44.2)	463
Norway (*N* = 17,092)					
Gender (%)	Women	49.1	73.4 (72.8, 74.1)	48.9 (47.8, 50.1)	12,550
	Men	50.9	26.4 (25.7, 27.1)	50.8 (49.6, 51.9)	4511
	Other	NA	0.2 (0.1, 0.3)	0.3 (0.2, 0.4)	31
Age groups (%)	18–34 years	31.5	35.0 (34.3, 35.7)	31.6 (30.6, 32.5)	5981
	35–54 years	38.0	49.5 (48.8, 50.3)	37.9 (36.9, 39.0)	8465
	≥55 years	30.5	15.5 (14.9, 16.0)	30.5 (29.2, 31.8)	2646
Educational attainment (%)	Primary	19.5	3.7 (3.4, 4.0)	15.0 (13.9, 16.2)	632
	Secondary	42.2	29.9 (29.2, 30.5)	46.6 (45.5, 47.8)	5102
	Higher	38.4	66.5 (65.7, 67.2)	38.4 (37.4, 39.3)	11,358
Spain (*N* = 3139)					
Gender (%)	Women	50.5	65.1 (63.4, 66.7)	50.4 (47.3, 53.6)	2043
	Men	49.5	34.8 (33.1, 36.5)	49.4 (46.3, 52.6)	1092
	Other	NA	0.1 (0.0, 0.3)	0.1 (0.0, 0.7)	4
Age groups (%)	18–34 years	25.0	31.7 (30.1, 33.3)	25.0 (22.4, 27.7)	994
	35–54 years	43.4	52.3 (50.6, 54.1)	52.4 (49.2, 55.5)	1642
	≥55 years	31.6	16.0 (14.8, 17.3)	22.7 (19.7, 25.9)	503
Educational attainment (%)	Primary	42.1	2.6 (2.1, 3.2)	11.1 (8.9, 13.7)	82
	Secondary	24.1	13.8 (12.7, 15.1)	55.2 (52.2, 58.1)	434
	Higher	33.7	83.6 (82.2, 84.8)	33.7 (31.5, 36.0)	2623

*Note:* NA, missing information.

As compared to the EUROSTAT population characteristics of the selected countries, the survey samples in these countries had a higher proportion of women, middle‐aged adults (35–54 years), and individuals with higher educational attainment, while people with primary or secondary education were underrepresented. After weighting, the gender, age, and education distribution matched the countries actual population in the case of Germany and Norway. In Czechia, matching the survey population to the actual population was successful with regard to gender and age, while individuals with a higher education were still overrepresented by about 10 percentage points, and those with secondary education were underrepresented by seven percentage points after the weights were applied. While the gender and education distributions of the Lithuanian sample were successfully approximated to the EUROSTAT population, young and middle‐aged adults remained overrepresented after weighting, whereas older adults were underrepresented by almost 17 percentage points. In Spain, the survey data could only be adjusted for the gender distribution. Middle‐aged adults remained overrepresented, and older adults underrepresented by almost nine percentage points. Additionally, individuals with primary education were underrepresented and those with secondary education underrepresented by more than 30 percentage points. The insufficient adjustment in Spain was caused by an imbalanced representation of women aged 35 and over, and of respondents with secondary and higher education (see Data S3, S4 for the distribution of sex, age, and education in the general population and the study sample). Modest adjustments after weighting in Czechia, Lithuania, and Spain resulted from a limited number of observations per gender‐age‐education strata. Due to the small number of observations, multiple strata needed to be collapsed, which in turn might have led to an insufficient adjustment to the population distribution. In the case of Spain, for example, each of two age and education groups in women needed to be combined to obtain weights below the threshold, yet this still did not guarantee sufficient matching with the population distribution.

## 4 DISCUSSION

In this paper, we have outlined different dissemination strategies, and their advantages and disadvantages for data collection via online surveys without external funding in five European countries. The strategies employed varied in terms of reach, response volume and data quality, as well as costs. While in some countries, strategies such as promotion by newspaper articles or paid social media advertisements led to high numbers of responses, the success of the dissemination strategies employed in other countries were more limited. Moreover, having a high number of respondents did not directly result in a representative sample of the actual population. Targeted sampling may help to improve representativeness, but this depends on time capacities and financial resources. While we were able to show that large‐scale general population samples can be obtained by means of online surveys in a short time, it is crucial to consider suitable strategies for each country.

Apart from these findings, attention needs to be paid to the established strengths and weaknesses of online surveys (Greenacre, 2016; Wright, 2006). While online surveys are a cost‐effective option to reach a high number of persons, they do not reach a target population beyond internet users. Thus, the extent to which the target population is covered depends on various factors, including the technical infrastructure and the level of internet use among relevant sub‐groups, such as elderly people or those with lower levels of education. Advantages of self‐selected surveys, such as the one we have used in this study, are their direct and easy access, and fully ensured anonymity which can facilitate self‐selection. However, self‐selected, non‐probabilistic surveys may lead to a biased sample that requires an application of statistical adjustments such as post‐stratification or weighting (Greenacre, 2016), as was done in our study. Additionally, sample bias may result from high non‐response rates (Rehm, Kilian, Rovira, et al., 2021). High non‐response (or non‐completion) rates are a major challenge in online surveys and may be partly due to break‐offs. Break‐offs can be particularly high in online surveys as the level of commitment is lower compared to personal or telephone interviews. In our study, only a third of the visitors to the survey link and three quarters of those who started the survey completed it. However, there are several methods that can be considered to reduce the likelihood of break‐offs (Mavletova & Couper, 2015). Such options to improve the survey design include a short survey length, less complex survey designs, or an adaptation to mobile online surveys (made available for different devices). With regard to mobile applications, however, it should be noted that a survey can take longer to complete on mobile devices than on personal computers (Couper & Peterson, 2017; Gummer & Roßmann, 2015) which may pose additional challenges (Mavletova & Couper, 2013). Moreover, the Norwegian experience shows the importance of ensuring sufficient server capacity when employing dissemination strategies that may attract a huge number of respondents within a narrow time frame.

Based on our experience and the literature, we propose the following approach to reach large‐scale samples using online surveys: First, set up an easy‐to‐access online survey avoiding obstacles to participation (such as a lack of anonymity), define a target sample and locate potential collaborators. Second, select dissemination strategies that are appropriate in your country and the target population, and implement them one by one in order to minimise the risk of technical problems. Thus, appropriate strategies for one country may not work for all countries or groups of people. For example, the website and social media posts of the Spanish National Drug Plan targeted mainly people with a higher level of education and, consequently, very few people with a lower educational attainment were reached through this channel. Third, it is important to monitor responses closely during the data collection phase, to identify under‐represented sub‐populations. This allows for adjustments of dissemination strategies to balance out skewed data as seen in the example of Germany. Fourth, sample weights should be calculated once the data collection has been completed, adjusting the sample surveyed to the actual population of the country. While this may improve the representativeness of the sample for the actual population, we would like to emphasise that achieving full representativeness is unlikely, as our recommendations presented here apply only within the known limitations of online surveys (see Greenacre, 2016; Mäkelä, 2021; Rehm, Kilian, & Manthey, 2021; Rehm, Kilian, Rovira, et al., 2021; Wright, 2006). Finally, the decision on the methodology and the consideration of online surveys should always depend on the study's objective.

Our project demonstrated that a sizable convenience sample could be obtained for a large number of European countries within a short time, if an active network exists or can be created. Further, this project was accomplished with a limited budget, since all other partner countries participated with no budget whatsoever or minimal financial resources. This does not mean that the efforts made in countries to translate and disseminate have no cost. At a time when most research is based on external funding and research institutions are organised to be ‘slim’, it is important that some staff and relevant infrastructure can be quickly deployed for a time‐limited effort to address research questions in exceptional situations. The success of our strategy underlines the need to further develop international cooperation in research in order to enable and facilitate activity of transnational projects. Such outstanding joint efforts should not be reserved for the exceptional situation of a public health crisis the size of a global pandemic, but should find their way into regular scientific practice.

## CONFLICTS OF INTERESTS

None.

## Supporting information

Supplementary Material

## Data Availability

Additional materials including the dataset are available in the Figshare repository, https://doi.org/10.6084/m9.figshare.13580693.v1.

## References

[mpr1875-bib-0001] Alkohol a COVID 19 . (2020, May 18). Portál Hlavrniho Města Prahy. Retrieved from https://www.praha.eu/jnp/cz/o_meste/magistrat/tiskovy_servis/Aktuality_archiv/alkohol_a_covid_19.html

[mpr1875-bib-0002] Braseth, S. (2020, May 20). Drikker vi mer under corona? Dagbladet. Retrieved from https://www.dagbladet.no/nyheter/drikker‐vi‐mer‐under‐corona/72480825

[mpr1875-bib-0003] Clay, J. M., & Parker, M. O. (2020). Alcohol use and misuse during the COVID‐19 pandemic: A potential public health crisis? The Lancet Public Health, 5(5), e259. 10.1016/S2468-2667(20)30088-8 32277874 PMC7195126

[mpr1875-bib-0004] Couper, M. P., & Peterson, G. J. (2017). Why do web surveys take longer on smartphones? Social Science Computer Review, 35(3), 357–377. 10.1177/0894439316629932

[mpr1875-bib-0005] EUROSTAT . (2020). DATASET: Population by educational attainment level, sex and age (1 000) (edat_lfs_9901). Retrieved from https://appsso.eurostat.ec.europa.eu/nui/show.do?dataset=edat_lfs_9901&lang=en

[mpr1875-bib-0006] Folkehelseinstituttet . (2020, May 20). Spørreundersøkelse om alkoholbruk under koronapandemien. Meldinger. Retrieved from https://www.fhi.no/meldinger/sporreundersokelse‐om‐alkoholbruk‐under‐koronapandemien/

[mpr1875-bib-0007] GBD 2017 Risk Factors Collaborators . (2018). Global, regional, and national comparative risk assessment of 84 behavioural, environmental and occupational, and metabolic risks or clusters of risks for 195 countries and territories, 1990‐2017: A systematic analysis for the global burden of disease study 2017. Lancet, 392(10159), 1923–1994. 10.1016/S0140-6736(18)32225-6 30496105 PMC6227755

[mpr1875-bib-0008] Greenacre, Z. A. (2016). The importance of selection bias in internet surveys. Ojs, 06, 397–404.

[mpr1875-bib-0009] Gummer, T., & Roßmann, J. (2015). Explaining interview duration in web surveys. Social Science Computer Review, 33(2), 217–234. 10.1177/0894439314533479

[mpr1875-bib-0010] Holmes, E. A., O'Connor, R. C., Perry, V. H., Tracey, I., Wessely, S., Arseneault, L., Ballard, C., Christensen, H., Cohen Silver, R., Everall, I., Ford, T., John, A., Kabir, T., King, K., Madan, I., Michie, S., Przybylski, A. K., Shafran, R., … Sweeney, A. (2020). Multidisciplinary research priorities for the COVID‐19 pandemic: A call for action for mental health science. The Lancet Psychiatry, 7(6), 547–560. 10.1016/S2215-0366(20)30168-1 32304649 PMC7159850

[mpr1875-bib-0011] Kilian, C. (2020a). Dissemination strategies 1.2. Figshare. 10.6084/m9.figshare.12738728

[mpr1875-bib-0012] Kilian, C. (2020b). Survey weights. Figshare. 10.6084/m9.figshare.12739469.v1

[mpr1875-bib-0013] Kilian, C. (2020c). Surveys. Figshare. 10.6084/m9.figshare.12738734.v1

[mpr1875-bib-0014] Kilian, C., Manthey, J., Braddick, F., Matrai, S., Gual, A., Rehm, J., & with the European Study Group on Alcohol Use and COVID‐19 (ESAC) . (2020). Changes in alcohol consumption since the outbreak of the SARS‐CoV‐2 pandemic in Europe: A study protocol. Retrieved from https://www.deep‐seas.eu/standard‐eu‐alcohol‐survey/

[mpr1875-bib-0015] Kruskal, W., & Mosteller, F. (1979). Representative sampling, III: The current statistical literature. International Statistical Review/Revue Internationale de Statistique, 47(3), 245. 10.2307/1402647

[mpr1875-bib-0016] LimeSurvey GmbH . (2020). LimeSurvey: An open Source survey tool. LimeSurvey GmbH. Retrieved from http://www.limesurvey.org

[mpr1875-bib-0017] Mäkelä, P. (2021). The future of alcohol surveys: Between the devil and the deep blue sea. Drug and Alcohol Review. dar.13162 40(2), 171–172. 10.1111/dar.13162 32959442

[mpr1875-bib-0018] Mavletova, A., & Couper, M. P. (2013). Sensitive topics in PC web and mobile web surveys: Is there a difference? Survey Research Methods, 7(3), 191–205. 10.18148/srm/2013.v7i3.5458

[mpr1875-bib-0019] Mavletova, A., & Couper, M. P. (2015). A meta‐analysis of breakoff rates in mobile web surveys. In D. Toninelli, R. Pinter & P. De Pedraza (Eds.), Mobile research methods: Opportunities and challenges of mobile research methodologies (pp. 81–98). Ubiquity Press. 10.5334/bar.f.License:CC-BY4.0

[mpr1875-bib-0020] Rehm, J., Gmel, G. E., Gmel, G., Hasan, O. S. M., Imtiaz, S., Popova, S., Probst, C., Roerecke, M., Room, R., Samokhvalov, A. V., Shield, K. D., & Shuper, P. A. (2017). The relationship between different dimensions of alcohol use and the burden of disease‐an update. Addiction, 112(6), 968–1001. 10.1111/add.13757 28220587 PMC5434904

[mpr1875-bib-0021] Rehm, J., Kilian, C., Ferreira‐Borges, C., Jernigan, D., Monteiro, M., Parry, C. D. H., Sanchez, Z. M., & Manthey, J. (2020). Alcohol use in times of the COVID 19: Implications for monitoring and policy. Drug and Alcohol Review, 39, 301–304. 10.1111/dar.13074. 10.1111/dar.13074 32358884 PMC7267161

[mpr1875-bib-0022] Rehm, J., Kilian, C., & Manthey, J. (2021). The future of surveys in the alcohol field. Drug and Alcohol Review. 40(2), 176–178. 10.1111/dar.13180 32959438

[mpr1875-bib-0023] Rehm, J., Kilian, C., Rovira, P., Shield, K. D., & Manthey, J. (2021). The elusiveness of representativeness in general population surveys for alcohol. Drug and Alcohol Review. 40(2), 161–165. 10.1111/dar.13148 32830351

[mpr1875-bib-0024] Uhlíková, M. (2020, May 28). Evropu zajímá naše spotřeba alkoholu v době koronaviru. IForm. Retrieved from https://iforum.cuni.cz/IFORUM‐16803.html

[mpr1875-bib-0025] World Health Organization . (2018). Global status report on alcohol and health 2018. World Health Organization. Retrieved from https://www.who.int/substance_abuse/publications/global_alcohol_report/en/

[mpr1875-bib-0026] Wright, K. B. (2006). Researching internet‐based populations: Advantages and disadvantages of online survey research, online questionnaire authoring software packages, and web survey services. Journal of Computer‐Mediated Communication, 10(3). 00–00 10.1111/j.1083-6101.2005.tb00259.x

