# Supplementary material for: Conducting a multi‐country online alcohol survey in the time of the COVID‐19 pandemic: Opportunities and challenges
Source: Int J Methods Psychiatr Res. 2021 May 5;30(3):e1875. doi: 10.1002/mpr.1875 (PMC8209885; doi:10.1002/mpr.1875)

# Supplementary material

**Supplementary material S1: Survey development**

# The basic questionnaire was developed in English and consisted of validated items (sociodemographic aspects, consumption items of the Alcohol Use Disorder Identification Test AUDIT-C (Bush et al., 1998)) and items developed for the purpose of the study (for details, see study protocol Kilian et al., 2020). These new items concerned (i) perception of measures to contain the spread of the COVID-19 pandemic, (ii) changes in alcohol consumption, and (iii) changes in the use of substances other than alcohol. Questions on changes in alcohol consumption followed the format of AUDIT-C items (frequency of drinking, quantity of alcoholic drinks per occasion, and frequency of heavy episodic drinking [HED] occasions), while changes in other substance use referred only to changes in the overall frequency of use. All questions related to the pandemic considered a time period of the past month (e.g., “Did you drink alcohol less or more often in the past month?”). The questionnaire was developed by an expert task force at the Technische Universität (TU) Dresden, Germany, and a first online pilot was tested by a number of reviewers, some of whom were experts in the field of alcohol survey research and some without such experiences.

# Once the final version of the questionnaire was in place (Kilian, 2020c), the text was gradually translated into other languages with a support from the existing network of the pan-European project *Developing and Extending Evidence and Practice from the Standard European Alcohol Survey* (DEEP SEAS), as well as from other health care professionals and alcohol researchers. The translation process was coordinated by the teams of TU Dresden (Germany) and Hospital Clínic Barcelona (Spain). Whenever a new country partner joined the project, they translated the original English questionnaire into their national language(s). Additionally, after the first surveys became publicly available, other researchers contacted our study group and asked to join in order to carry out the survey in their country. By mid-May, three weeks after launching the project, 21 translations of the survey were available. The questionnaires in all languages were incorporated into the open-source online tool LimeSurvey (LimeSurvey GmbH, 2020), and a pilot was coordinated by the partner in each country. After the final revision, each different language version of the questionnaire was published and hence publicly available on our study homepage ([www.covid19-and-alcohol.eu](http://www.covid19-and-alcohol.eu)). The survey was distributed in a decentralized manner under the responsibility of the national partners. Detailed information on the dissemination strategies used in every country are published online (Kilian, 2020a).

**Supplementary material S2: Dissemination strategies**

| **Country** | **Dissemination period** | | **Professional networks** | **Mailing lists** | | **Website and social media posts** | | | **Media** | **Paid**  **facebook ad** | **Support of ministry of health** |
| --- | --- | --- | --- | --- | --- | --- | --- | --- | --- | --- | --- |
|  | **Start date** | **End date** |  | **Students** | **Others** | **Institution** | **Facebook** | **Other social media** |  |  |  |
| **Albania** | 27.04.20 | 30.06.20 | x | x |  | x |  | x |  |  |  |
| **Czechia** | 24.04.20 | 30.06.20 | x | x | x | x | x |  | x | 0 EUR | x |
| **Denmark** | 14.05.20 | 01.07.20 | x |  | x | x | x |  |  | 300.00 DKK (≈ 40 EUR) |  |
| **Finland** | 25.05.20 | 30.06.20 | x |  | x | x | x | x |  | 200.00 EUR |  |
| **France** | 29.04.20 | 22.07.20 | x |  |  | x | x |  |  | 40.00 EUR |  |
| **Germany** | 24.04.20 | 30.06.20 | x | x |  | x |  | x |  | 500.00 EUR |  |
| **Greece** | 22.06.20 | 22.07.20 | x | x | x |  | x | x |  | 0 EUR |  |
| **Hungary** | 01.07.20 | 16.07.20 | x |  | x | x | x |  |  | 0 EUR |  |
| **Iceland** | 24.04.20 | 30.06.20 | x |  |  | x | x |  |  | 65.00 EUR |  |
| **Ireland** | 01.05.20 | 30.06.20 | x | x | x | x | x | x | x | 200.00 EUR |  |
| **Italy** | 25.04.20 | 30.06.20 | x | x | x | x | x | x | x | 0 EUR |  |
| **Norway** | 29.04.20 | 30.06.20 | x |  |  | x | x |  | x | 0 EUR |  |
| **Poland** | 29.04.20 | 30.06.20 | x |  | x | x | x |  |  | 0 EUR |  |
| **Portugal** | 25.04.20 | 30.06.20 | x |  | x |  |  |  |  | 0 EUR | x |
| **Russia** | 12.05.20 | 30.06.20 | x | x |  | x | x |  | x | 0 EUR |  |
| **Slovakia** | 17.05.20 | 30.06.20 | x | x | x | x | x |  | x | 0 EUR |  |
| **Slovenia** | 15.05.20 | 30.06.20 | x |  | x | x | x |  |  | 0 EUR | x |
| **Spain** | 25.04.20 | 30.06.20 | x |  |  |  |  |  |  | 0 EUR | x |
| **Sweden** | 20.05.20 | 30.06.20 | x | x | x |  | x |  |  | 0 EUR |  |
| **Ukraine** | 30.04.20 | 30.06.20 | x |  |  |  |  |  |  | 0 EUR |  |
| **UK** | 25.04.20 | 31.07.20 | x |  | x |  | x | x |  | 210.00 GBP (≈ 233 EUR) |  |
| Note. UK = United Kingdom. | | | | | | | | | | | |

Table S1. Overview of dissemination strategies employed by country (first survey wave only).

**Supplementary material S3: Comparison of the actual European population with the unweighted and weighted survey population by gender and age**

Figure S1. Comparison of the actual European population with the unweighted and weighted survey population by group of age, among men.


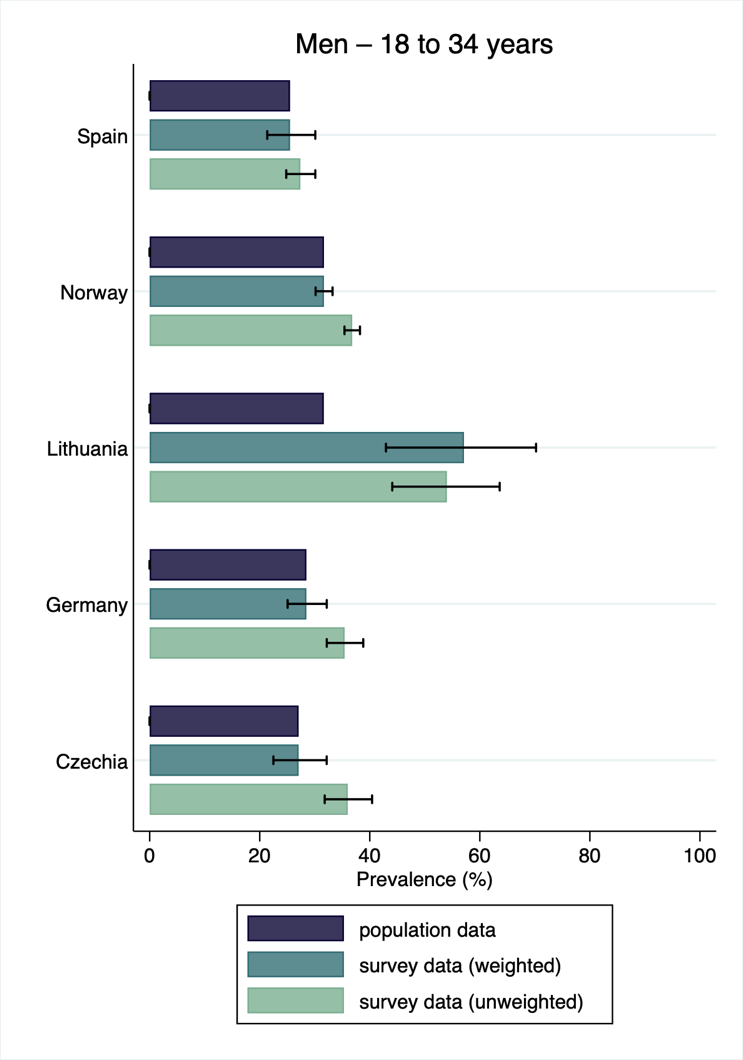

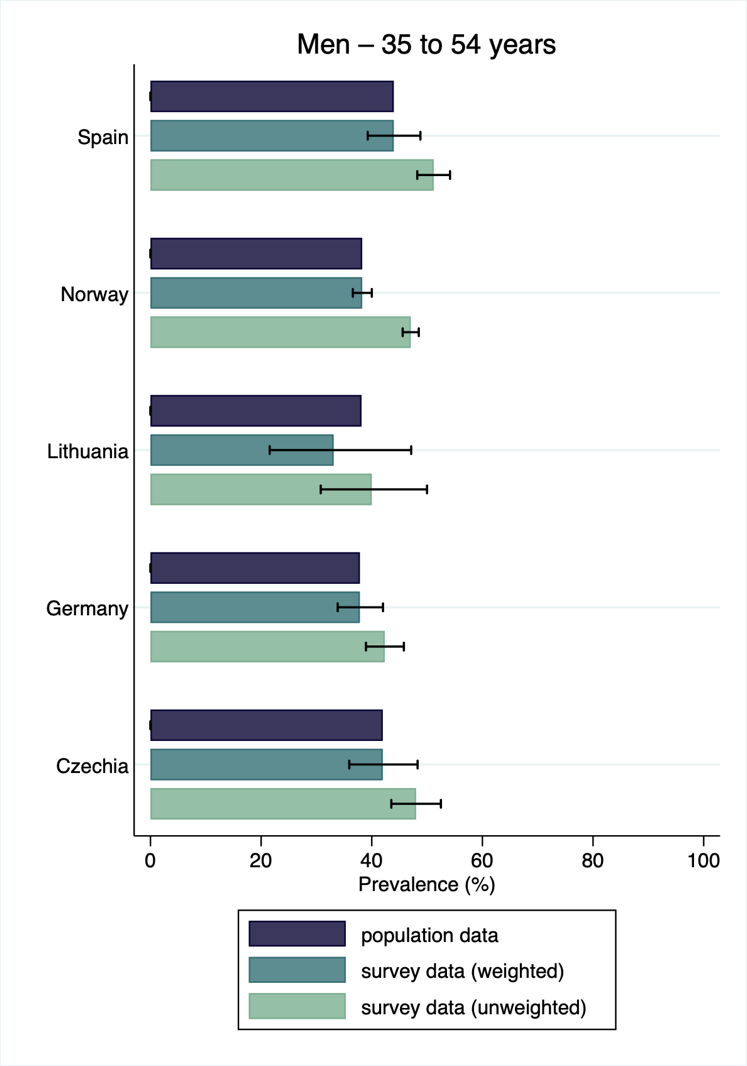

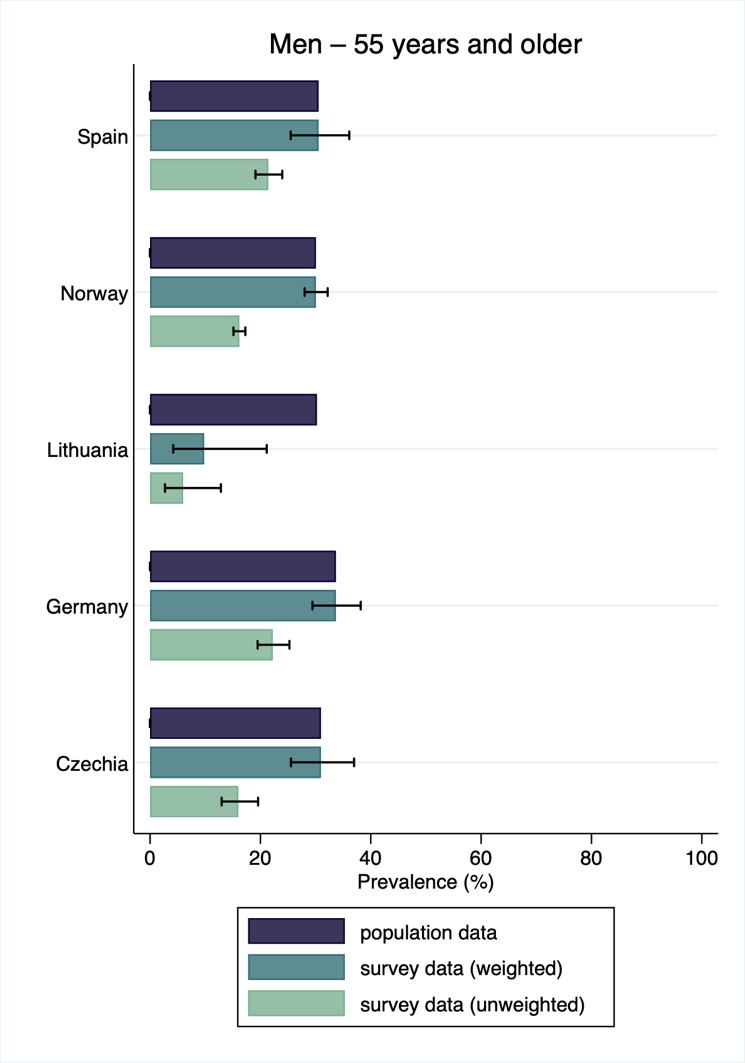

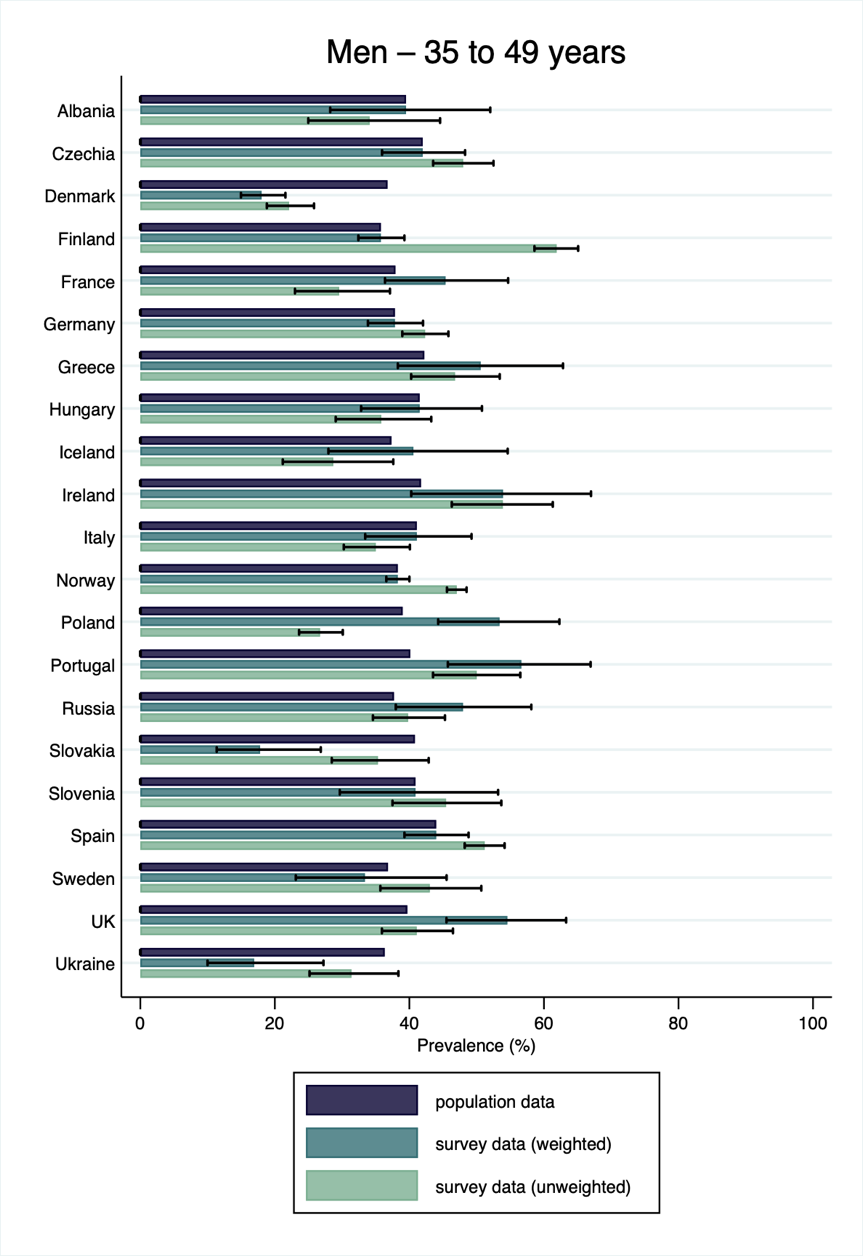


Figure S2. Comparison of the actual European population with the unweighted and weighted survey population by group of age, among women.


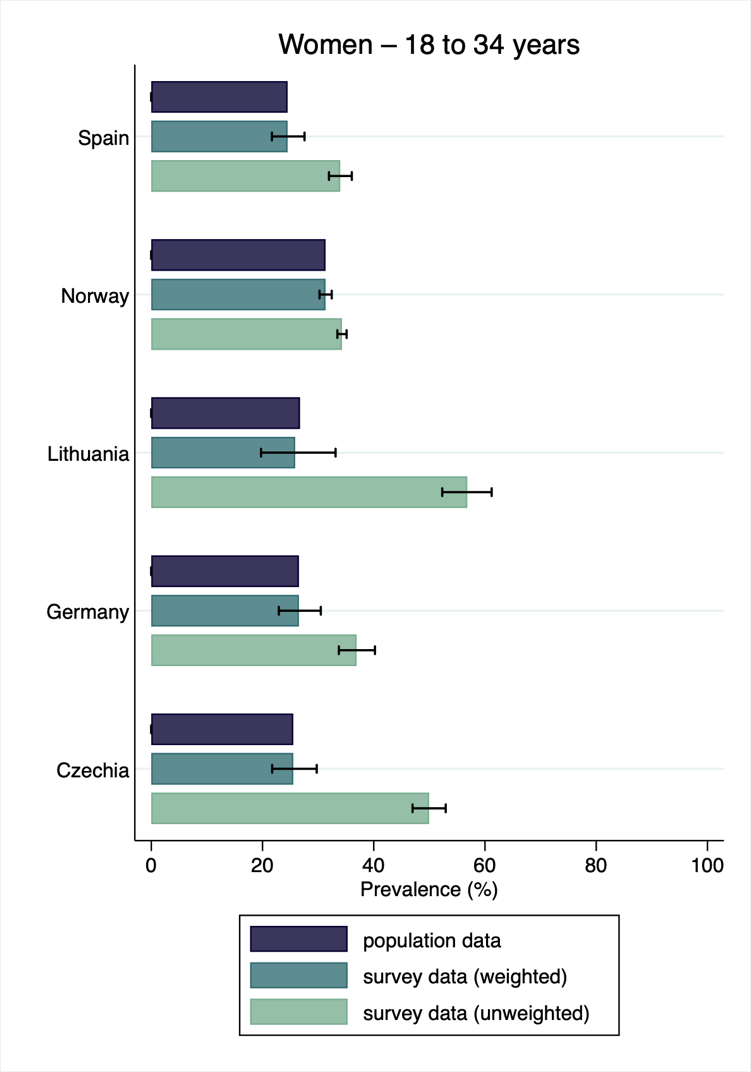

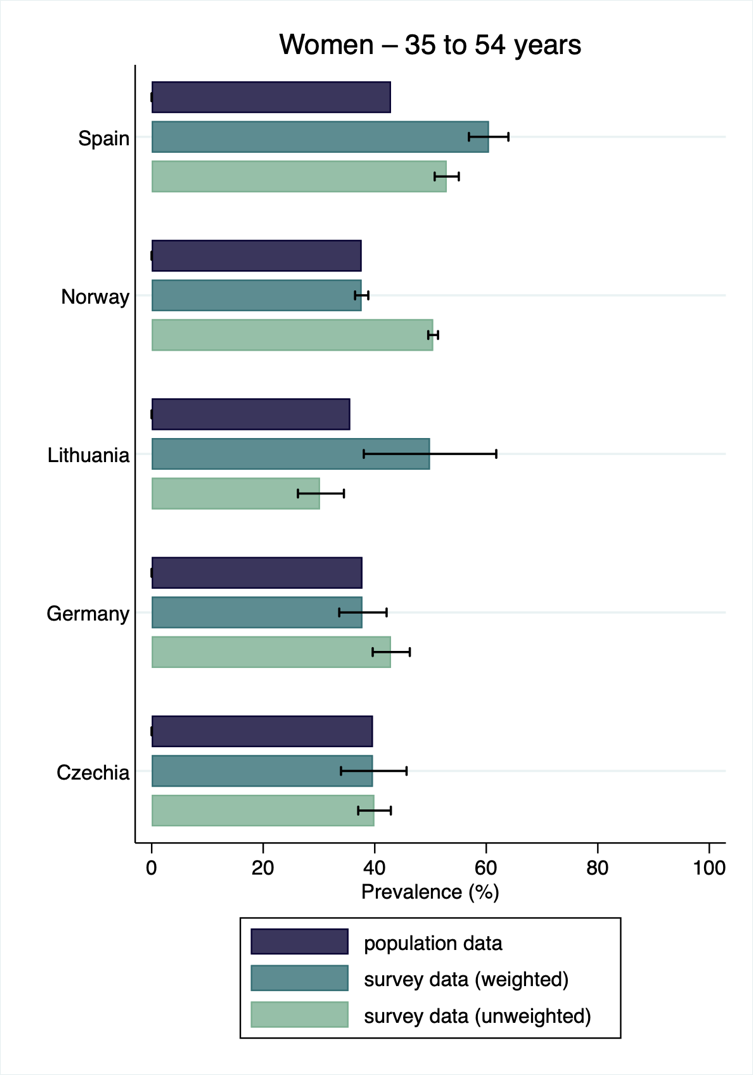

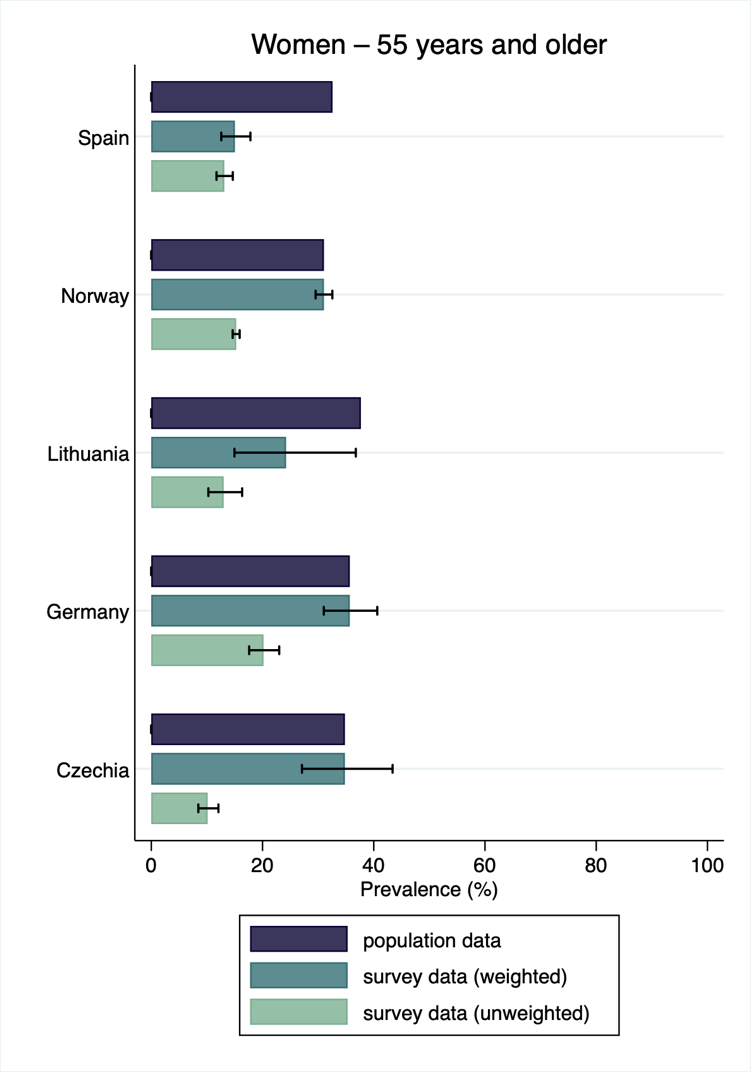

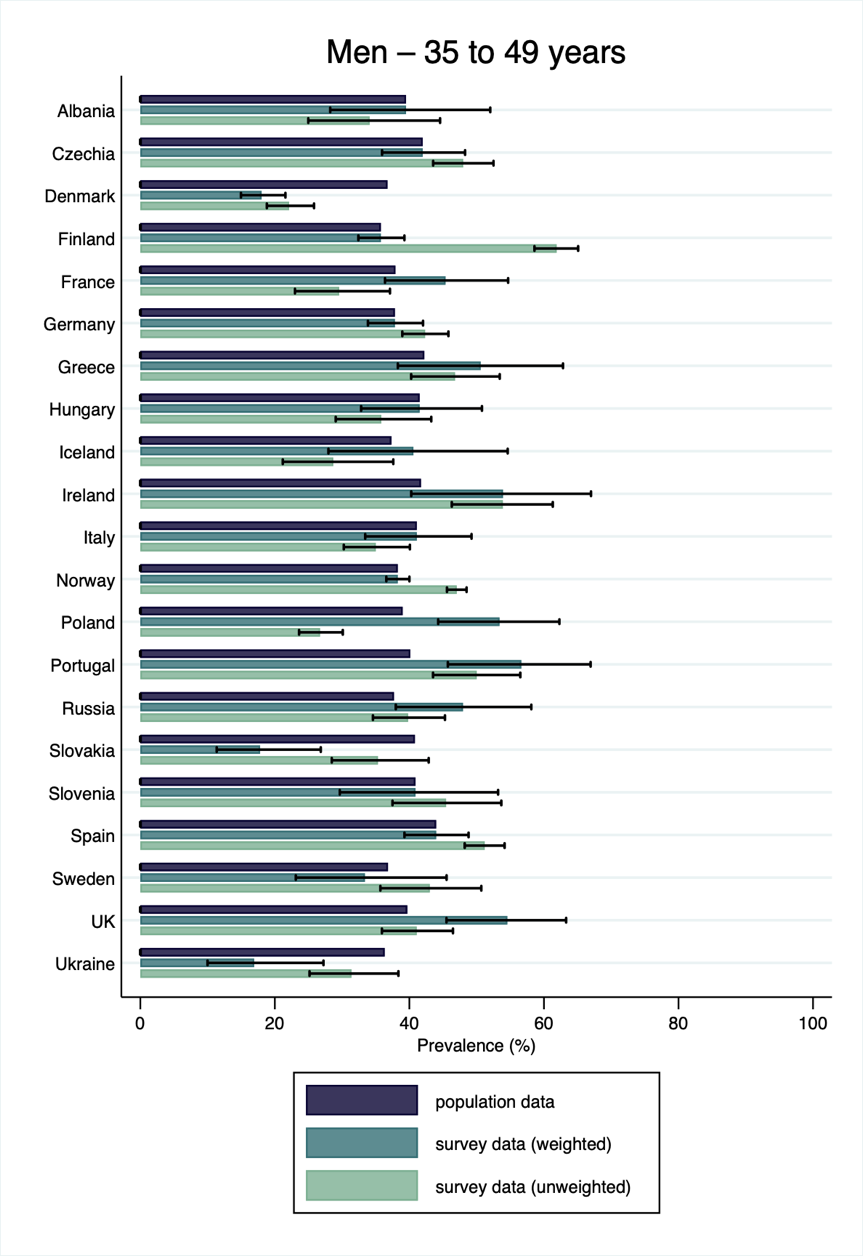


**Supplementary material S4: Comparison of the actual European population with the unweighted and weighted survey population by educational attainment**

Figure S3. Comparison of the actual European population with the unweighted and weighted survey population by educational attainment for both, women and men.


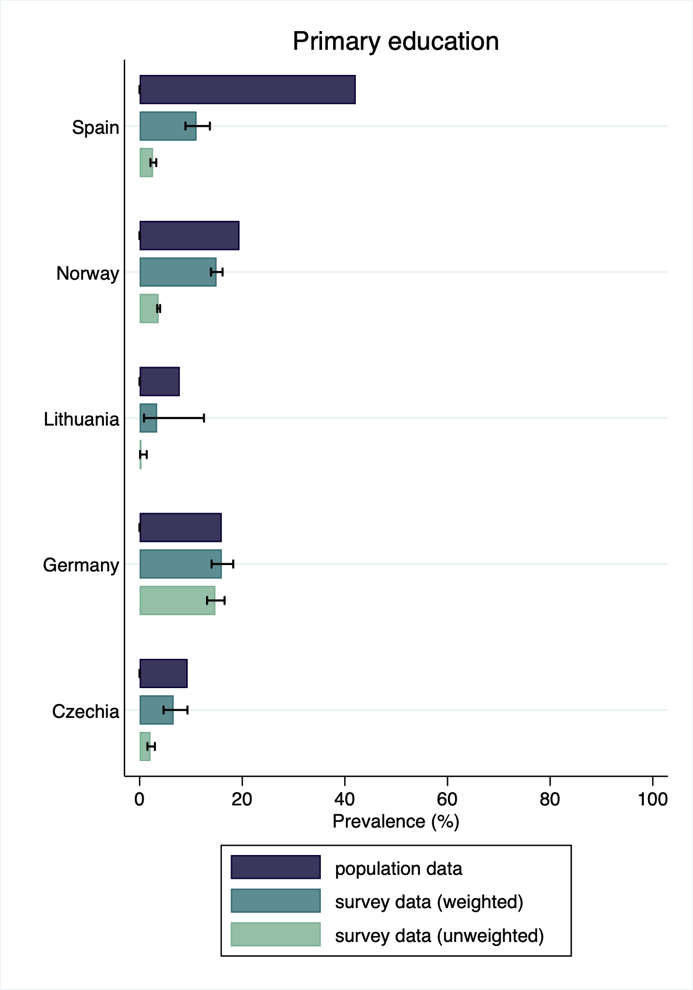

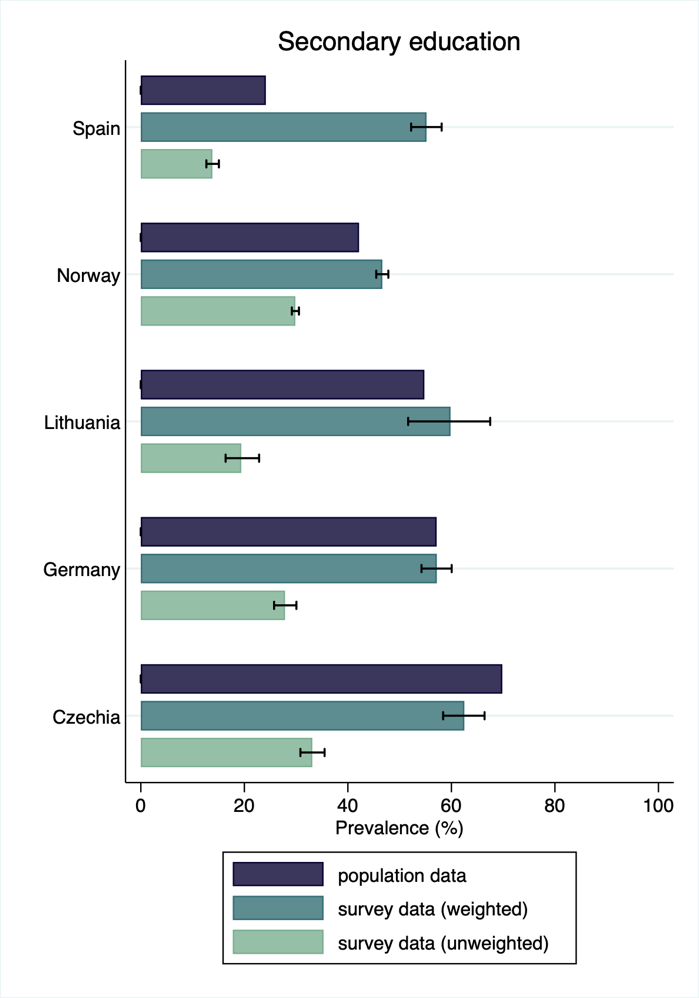

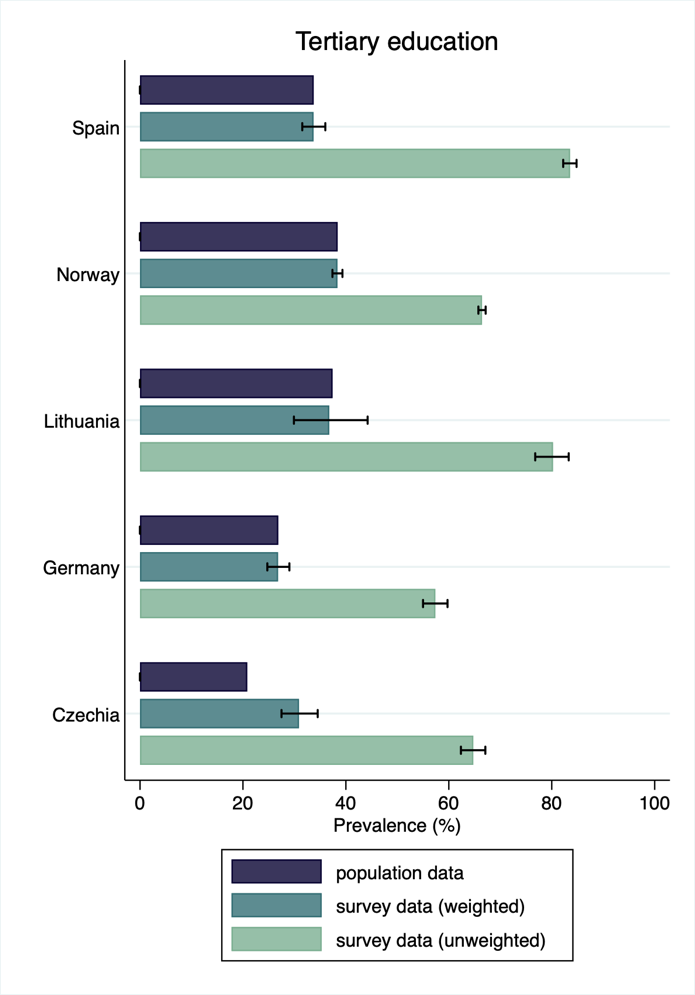

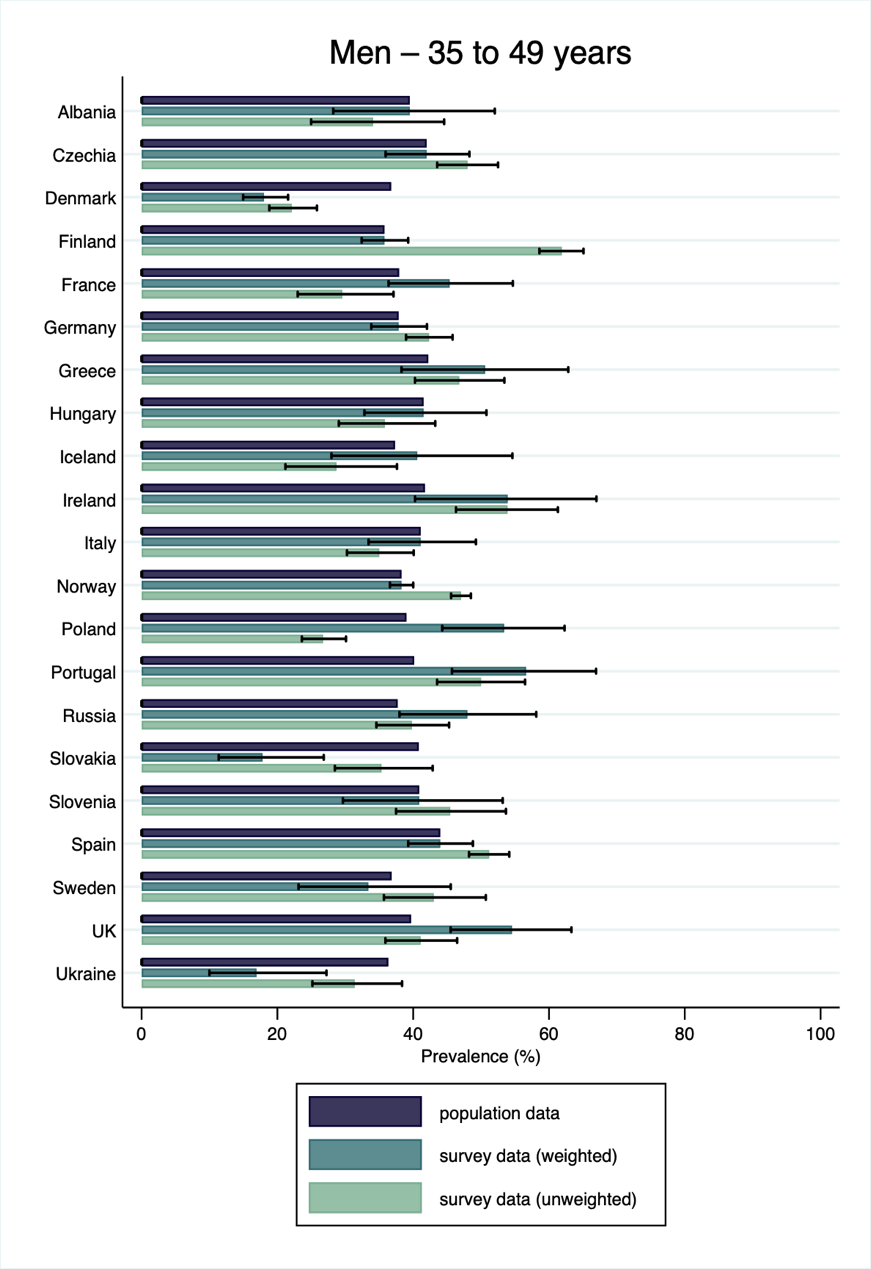

Supplement: Supplementary file 1 — Supplementary Material [file MPR-30-e1875-s001.docx]
